# Bitter silence allows sexual harassment to continue in workplace: A qualitative study in Iranian nurses

**DOI:** 10.3389/fpubh.2022.971522

**Published:** 2022-09-12

**Authors:** Maryam Zeighami, Mohammad Ali Zakeri, Parvin Mangolian Shahrbabaki, Mahlagha Dehghan

**Affiliations:** ^1^Department of Nursing and Midwifery, Kerman Branch, Islamic Azad University, Kerman, Iran; ^2^Determinants of Health Research Centre, Non-Communicable Diseases Research Center, Rafsanjan University of Medical Sciences, Rafsanjan, Iran; ^3^Department of Critical Care Nursing, Razi Faculty of Nursing and Midwifery, Kerman University of Medical Sciences, Kerman, Iran

**Keywords:** sexual harassment, nurse, disclosure, content analysis, Iran

## Abstract

**Background:**

Sexual harassment in the workplace is continuing. However, the rate of sexual harassment disclosure is low, which causes many problems. Non-disclosure of sexual harassment can cause nurses' psychological distress and physical harm, and affect their productivity and quality of care. Therefore, the present study aimed to investigate the reasons why Iranian nurses stayed silent and did not disclose sexual harassment in their workplace.

**Method:**

This qualitative descriptive-explorative study was conducted to investigate the reasons why Iranian nurses (*n* = 18) stayed silent on sexual harassment. Conventional content analysis and purposeful sampling method were used in this study. Data was collected through in-depth semi-structured interviews. Maximum variance in terms of age, sex, work experience, education level, marital status, and type of hospital and ward was considered in order to obtain rich information. Guba and Lincoln criteria were used to increase the study's trustworthiness, while the Graneheim and Lundman approach was used to analyze the content.

**Results:**

The research data indicated 112 codes, a major category, 4 subcategories, and 9 primary categories. The major category, “The missing link is bitter silence; sexual harassment is still going on,” includes four subcategories: fear of social stigmas, organizational and legal barriers, family barriers, and personal barriers.

**Conclusions:**

Nurses cannot break their silence on sexual harassment because they are afraid that disclosure of sexual harassment has negative consequences for their personal and professional lives. Policies and strategies should be developed to encourage nurses to disclose sexual harassment. This issue must be studied socially, culturally, and politically.

## Introduction

Violence against healthcare workers is a global phenomenon that appears to be increasing at an alarming rate (1). Compared to other health workers, nurses have been the most targeted by violence worldwide (2). Several studies have shown that more than half of nurses have experienced verbal assault and about a third have experienced physical assault (3). Yang et al. stated that 94.6% of nurses reported a high prevalence of workplace violence, from verbal violence and physical assault to sexual harassment (4).

Sexual harassment is known as unpleasant and annoying sexual behaviors, including verbal, physical, psychological, and visual types that are common in the workplace (5) and are associated with insulting, humiliating, and threatening the health of the victims (6). Sexual harassment is the coercion of a person, regardless of their consent, which occurs in a context where power relations are unequal (7).

Studies show that sexual harassment is widespread in workplaces and has a different prevalence in different countries (8). More than half of the nurses in the nursing system worldwide have experienced sexual harassment (9). Kahsay et al. (10) stated that 10–87.3% of nurses have experienced sexual harassment. The prevalence of sexual harassment among nurses in Asia is 21.6%, Europe at 16.2%, the Middle East at 22.4%, England at 38.7% (2), China at 3.9% (11), Malaysia at 51.2% (6), and Iranian nurses 1.07–9.5% have been reported (12).

However, the results of a study on the prevalence of sexual harassment cover a wide range. The reason for these differences is probably related to the tools used, the sampling method, and differences in the social, cultural, and economic environment. Thus, the findings of one country cannot be generalized to other countries (5).

Sexual harassment occurs globally in the nursing profession and challenges nurses' health and safety, as well as the quality and efficiency of healthcare systems. Some reports indicate that sexual harassment in the workplace is one of the major concerns for nurses (13). Sexual harassment has many negative consequences for nurses' personal and professional lives and imposes a heavy burden on the healthcare system because it reduces productivity and the active labor force (14). Sexual harassment makes nurses feel embarrassed or humiliated, reduces their ability to do the right thing, and compromises their wellbeing by reducing their quality of professional life. In addition, sexual harassment in hospitals reduces proper nursing care and thus affects patient care (8, 15). Ali and Ezz El Rigal (16) showed a high rate of sexual harassment in clinical settings, which affected nurses' lives, leading to poor practice and burnout.

An important issue that emphasizes the importance of paying attention to sexual harassment in nurses is that many incidents of sexual harassment in the workplace are not reported due to cultural factors, negative consequences, peer pressure, and the victim's unwillingness. Many victims believe that reporting an incident is pointless, or because of previous experiences or even a lack of knowledge of policies, prefer to remain silent and refuse to report (17). Social, economic, and cultural contexts and norms affect sexual harassment as a subjective experience (18, 19). Women will face social stigma, humiliating statements, and discrimination if they act against their social roles in a given culture. As a result, women in some countries avoid breaking traditional norms, and instead of fighting against sexually abusive behavior, they choose to ignore it in order to maintain their status as respectable women. Thus, sexual harassment has become socially acceptable (20), which can affect the experience and reporting of sexual harassment. Therefore, many women do not disclose their sexual harassment to anyone unless they have experienced severe abuse (21). Fear of reporting and stigma attached to the victim must be eliminated. Leaders and managers must recognize sexual harassment among nurses, promote the values and standards that shape behaviors, decisions, and relationships, instill an ethical organizational culture, and provide a safe environment (13). Sexual harassment is illegal and should be considered by managers because of its very destructive effects on individuals. Organizations, employers, and institutions are expected to protect employees against sexual harassment in the workplace (22).

Some studies have provided precise and rapid information on violence in the nursing system. The high rate of physical violence is well known and has been the focus of workplace interventions in many organizations, but the high rate of other violent behaviors, such as sexual harassment, may be less acknowledged (2). Failure to report is a significant obstacle to eradicating this phenomenon from the nursing system. Nurses feel uncomfortable sharing the incidents they have encountered (23). Workplace problems and conditions should ensure adequate preventive interventions and an effective workforce. First, the problems of non-reporting should be addressed to understand and prevent sexual harassment. Current gaps in research on non-reporting constitute a significant obstacle to a better understanding of sexual harassment. Since quantitative research methods cannot comprehensively address such issues, qualitative research is used to explore phenomena in depth. Therefore, examining nurses' experiences, statements, and feelings about their silence on sexual harassment is necessary. This study helps to fill in some of these gaps by looking at why Iranian nurses in Kerman hospitals don't talk about sexual harassment.

## Materials and methods

### Study design and setting

This qualitative descriptive-explorative research used conventional content analysis to explore indivuduals' understanding of daily life phenomena and to interpret the content of subjective data. Explicit and implicit concepts are identified based on the participants' descriptions, which can be coded, condensed, categorized, and then themes are extracted. Codes are extracted based on meaning units derived from participants' descriptions and then categorized based on differences or similarities (24). This study tried to analyze implicit and explicit content. This study was conducted in Kerman, the largest city in southeastern Iran.

### Sampling, participant, and data collection

Purposive sampling with maximum variance was used to select participants. First, female nurses working in different wards of hospitals affiliated to Kerman University of Medical Sciences (KUMS) were interviewed. In addition, nurses working in private hospitals must be interviewed. Data saturation was obtained after 18 interviews. Various personal and occupational characteristics, such as age, marital status, education level, work experience, position, hospital type (public, private, educational), and hospital wards, were selected to provide a wide range of information. Nurses were between 25 and 51 years old and had 2–28 years of work experience (Table 1). Participants were selected based on some inclusion criteria. Nurses who had bachelor's or higher degrees and clinical experience were interviewed in this study. The first researcher conducted semi-structured and face-to-face interviews. Table 2 shows some of the questions reflecting participants' reasons for silence on sexual harassment. The interviews lasted 30–100 mins, were audio recorded, and then were transcribed verbatim. Sampling was performed from September 2020 to August 2021.

**Table 1:** Participants' characteristics (*N* = 18).

	**Participants**	**Number**
Age (year)	Minimum	25
	Maximum	51
Marital status	Single	4
	Married	11
	Divorced	3
Work experience (year)	Minimum	2
	Maximum	28
Education level	Bachelor's	11
	Master's	6
	PhD	1
Employment status	Contract recruiters^a^	4
	Contract recruiter^b^	2
	Hired	12
Position	Nurse	10
	Head nurse	2
	Supervisor	3
	Faculty member	3
Type of hospital*	Educational-public	16
	Non-educational-public	1
	Private	6
Ward type*	Emergency	8
	Medical surgical	4
	ICU	5
	Operation room	4
	CCU	4
	General surgery	4
	Nursing management office	3
	Orthopedics	2
	Psychiatrics	3
	Pediatrics	1
	Gastro-intestinal	2
	Burn	1
	Dialysis	1

^*^ Some participants had work experience in different hospitals and wards.

^a^ Annually contracted with payment less than hired nurses. ^b^ Annually contracted with payment similar to hired nurses.

**Table 2:** Example of questions.

**Questions**
1. Would you please share your experience of sexual harassment in your workplace?
2. Did you share this experience with anyone or did you disclose it to the authorities?
3. What made you remain silent on sexual harassment?

### Data analysis

Data was collected and analyzed simultaneously. Recorded interviews were transcribed. As the researcher must immerse herself in data, she listened to the interviews several times and reviewed the manuscript several times. She divided the text into meaning units. Then, she condensed the meaning units while maintaining the theme. She labeled condensed meaning units with a code and created subcategories. Next, she created the categories that were the major characteristics of qualitative content analysis (25). In the present study, the major category “bitter silence that allows sexual harassment in the workplace to continue” was obtained. Although the analysis process was systematic, the researchers had to be able to move back and forth between the whole and the segments of the text. Table 3 provides an overview of the analysis process performed on each text. Table 4 provides an overview of all subcategories and primary categories. The analysis process lasted from September 2020 to September 2021.

**Table 3:** Example of qualitative content analysis process.

**Meaning unit**	**Condensation**	**Code**	**Subcategories**	**Categories**	**Main category**
When I was accepted as a nursing student, the public attitude toward nursing was negative. Even my father requested me not to enter this profession because he believed that hospital environment was not morally good, and the public attitude toward nursing was negative and stigmatic	Negative attitudes of parents and society toward nursing profession, and avoding children to enter this profession	Negative attitude of the family due to nurses' social stigma	Stigma toward the nursing profession	Fear of social stigmas	The missing link is bitter silence; Sexual harassment is still going on
What should I do? Whom should I talk to, and how could I prove it? I might be fired. I need this job. It is very difficult to be harassed and silent, while also being blamed by others.	Nurses did not inform authorities of their sexual harassment because they could not prove it and might be fired.	Silence on sexual harassment due to fear of losing job	Fear of work instability	Organizational and legal barriers	
I am afraid that if I disclose it, my husband will become aware of it. My husband repeatedly warned me that if someone at work bothered me, he would not let me go to work. I am afraid of losing the job for which I studied and worked so hard.	Nurses were silent on sexual harassment because they were afraid that their husbands would become aware of it and confine them.	Nurses were afraid that their husbands would find out and refuse to let them go to wrok.	Fear of having a more confined independent mobility by the family	Family barriers	
He touched me in a private room. I could not get away from him. I could not scream because I was afraid that others would find out and I would be humiliated.	The nurse was touched and was unable to react due to fear of being disgraced.	Silence on sexual harassment due to fear of losing honor	Fear of being accused and disgraced	Personal barriers	

**Table 4:** Main category, categories, and subcategories extracted from qualitative content analysis.

**Main category**	**Categories**	**Subcategories**
The missing link is bitter silence; sexual harassment is still going on	Fear of social stigmas	- Stigma toward the nursing profession - Negative attitudes toward women's independent living
	Organizational and legal barriers	- Weak legislation and prevention policies - Lack of support from authorities - Fear of work instability
	Family barriers	- Fear of having a more confined independent mobility by the family - Fear of being blamed by the family
	Personal barriers	- Fear of being accused and disgraced - Loss of trust in others

### Trustworthiness

**T**he study's trustworthiness was evaluated based on four criteria proposed by Lincolon and Guba, including credibility, confirmability, dependability, and transferability (26). Several methods have been used to increase the study's trustworthiness. Two research observers conducted the peer checking. The researcher and observers reported and discussed the research progress and process through several meetings. Member checking was conducted with some participants to confirm the interpreted findings (codes and categories). Some faculty members reviewed the coding process and code acquisition (external check). In addition, it was also very clear how the participants were chosen and how the data was collected and analyzed.

### Findings

This content analysis explains and defines the meaning, dimensions, and components of “The missing link is bitter silence; sexual harassment is still going on.” The findings of this study were extracted into the forms of a main category, four categories, and nine subcategories. In addition, 112 codes remained after continuous comparative analysis, code condensation, and integration (Table 4).

#### Main category

The missing link is bitter silence; sexual harassment is still going on according to the participants, this main category includes four subcategories: fear of social stigmas, organizational and legal barriers, family barriers, and personal barriers.

### Fear of social stigma

Nurses participating in the study did not break their silence on sexual harassment due to the fear of social stigmas, which occurred in the form of stigma toward the nursing profession and a negative attitude toward women's independent living.

#### Stigma toward the nursing profession

According to most participants in the study, one of the reasons for the silence on sexual harassment was the stigma toward the nursing profession. Participants believed that this negative attitude and stigma started from the time they entered the university and increased in their workplace. This negative attitude and stigma came from their families, spouses, friends, acquaintances, and the community. In addition, physicians have abused nurses because of their domination over nurses as well as nurses' low level of education.

“*My workplace was directly across from my husband's office, and he did not want any of his colleagues to know I was a nurse, because his colleagues had a negative attitude toward nurses.” (Participant No. 14, a nurse with 28 years of clinical and educational work experience)*

#### Negative attitudes toward women's independent living

Some study participants were living independently for reasons such as being away from their families, the death of, or separation from their spouses. They reported that their male colleagues and physicians abused them. Even their female colleagues had negative attitudes toward them, so they preferred to stay silent about their independent lives and sexual harassment.

“*One of the specialists, who was aware of my separation from my husband, was looking for an opportunity to abuse me. Although I could raise this issue with the hospital's security, they would likely blame me, so I chose to stay silent.” (Participant No. 5, a nurse with 24 years of clinical work experience)*

#### Organizational and legal barriers

Nurses participating in the study did not break their silence on sexual harassment due to organizational and legal barriers that occurred for the following reasons: weak legislation and prevention policies; lack of support from authorities; and fear of work instability.

#### Weak legislation and prevention policies

According to most participants in the study, the silence on sexual harassment was due to a lack of policymaking and legislation. Participants acknowledged that the lack of a protocol for reporting and registering sexual harassment, a lack of a reference for addressing complaints, a lack of supervision on sexual harassment complaints, a lack of legal strategies and supportive laws, or being unaware of the complaint process and legal law were the reasons for their silence on sexual harassment.

“*When a doctor harassed me, I informed the head nurse that I was going to file a complaint and asked her to assist me. She informed me that there was no law in this regard and that no one supported me, so I chose to remain silent.” (Participant No. 6, a nurse with 2 years of clinical and educational work experience)*

#### Lack of support from authorities

Some participants in the study remained silent on sexual harassment because the authorities were unwilling to accept it, were indifferent in this regard, and did not support sexually harassed individuals.

“*A patient was harassing me, and I reported it to the nurse manager. He explained to me that these were common incidents and that I would have to deal with them. However, dealing with such issues was illogical for me.” (Participant No. 12, a 27-year-old female nurse with 4 years of clinical work experience)*

#### Fear of work instability

According to some of the nurses, when doctors sexually harassed nurses, they remained silent due to fear of losing their position or being fired. In some cases, they had to remain silent in order to promote their position.

“*One of the physicians insisted that I accompany him during his visit. He was very relaxed and made sexual jokes. He wrapped his hand around my waist and touched me; I could not say anything because it was a private hospital and I was about to lose my job.” (Participant No. 9, a nurse with 4 years of clinical work experience)*

### Family barriers

Nurese in this study reported that they were silent on sexual harassment due to family barriers that occurred for the following reasons: fear of being more confined to independent mobility by their family; fear of being blamed by their family.

#### Fear of having a more confined independent mobility by family

Some nurses in the study did not break their silence on sexual harassment in the workplace because they were afraid that their parents or spouses would confine their independent mobility or even prevent them from going to work.

“*I do not want my husband to know because he is very sensitive about it. If he discovered what happened to me in the hospital, he would not let me leave the house.” (Participant No. 1, a nurse with two years of clinical work experience)*

#### Fear of being blamed by family

Some nurses in the study did not disclose sexual harassment in their workplace because they were afraid of being blamed by their families. In these cases, families did not support them and solve the problem, and they even blamed them, so nurses preferred to remain silent in similar cases.

“*I informed my sister of what had happened in the hospital. I expected her to understand me, but she immediately began blaming me. I would prefer to remain silent and avoid explaining these incidents to anyone.” (Participant No. 13, a nurse with eight years of clinical work experience)*

### Personal barriers

Nurses participating in the study remained silent on sexual harassment due to personal barriers that occurred in the following ways: fear of being accused and disgraced; and loss of trust in others.

#### Fear of being accused and disgraced

Most nurses in the study chose to remain silent due to cultural issues and fear of being accused and judged by others.

“*I was powerless to react to it because I knew I would humiliate myself in front of everyone else. I was unable to inform anyone that a doctor had touched me.” (Participant No. 2, a nurse with 26 years of clinical work experiences)*

#### Loss of trust in others

Some nurses in the study remained silent on sexual harassment because they did not trust others. They were afraid that talking about this incident, even with close friends, would make the situation worse.

“*I no longer trusted anyone because I was afraid that my close friend would tell everyone about what had happened.” (Participant No. 13, a nurse with eight years of clinical work experience)*

## Discussion

The present qualitative study was conducted to examine the reasons why Iranian nurses did not break their silence on sexual harassment in the workplace. According to this study, nurses' reasons for silence on sexual harassment should be paid much more attention to. The present study showed that nurses refused to disclose workplace sexual harassment because of (1) fear of social stigmas, (2) organizational and legal barriers, (3) family barriers, and (4) personal barriers.

### Fear of social stigmas

Nurses participating in the study chose to remain silent on sexual harassment due to the fear of social stigmas. Furthermore, Tollstern Landin et al. (27) in Tanzania, Africa, showed that the consequences of workplace sexual harassment could be a serious occupational risk for nurses. Victims of sexual harassment felt ashamed and angry, and they were reluctant to return to work (27). Adikaram et al. (18) in Sri Lanka indicated that the social construction of gender and sexuality in Sri Lankan society, with its instilled moralistic beliefs and norms such as respectability, sexual innocence, chastity, and purity among women, suppressed and governed their sexuality in the workplace. Adams et al. (13) in Sri Lanka also found that sexual harassment in the workplace led to negative social attitudes toward the nursing profession. Ross et al. (23) reported that sexual harassment was increasingly identified as an issue of gender, roles, power, society, and organizational demographics. The fear of reporting and the stigma attached to victims must be eliminated. Some nurses attributed their inaction to shyness, fear, and blame. The impact of social norms on victim blame, power, job security consequences, feelings of shame, and other potentially negative consequences are among the factors that reduce reports of sexual harassment (28). Sexual harassment is a major concern for nurses, so they must break their silence. To get a more complete picture, more research needs to be done to find out how bad the effects of sexual harassment are and how vulnerable the victims are.

### Organizational and legal barriers

Nurses participating in the study reported organizational and legal barriers as one of the reasons for their silence on sexual harassment. Ross et al. (23) believed that practical recommendations were necessary for healthcare organizations and nurses to reduce sexual harassment. Developing zero tolerance policies, modeling appropriate behaviors, and empowering nurses were essential to eradicate sexual harassment in workplace (23). Adikaram et al. (18) in Sri Lanka found that sexual harassment policies and practices should address the gender realities of women in the workplace and avoid using sexual stigma in the workplace. The role and function of healthcare institutions to prevent sexual harassment includes political, educational (educational institutions in healthcare), and organizational dimensions (22). Therefore, organizations have attempted to discover how sexual harassment develops and how to develop prevention policies. As sexual harassment in the workplace has led to negative social attitudes toward the nursing profession, employers must be careful to prevent sex in the workplace and correct sexual harassment as soon as possible (29). Organizations must try to prevent negative social attitudes and implement more policies to address this problem. In addition, healthcare organizations must develop zero-tolerance policies, model appropriate behaviors, and empower nurses to eradicate sexual harassment in the workplace (13).

### Family barriers

Nurses participating in the study did not disclose sexual harassment due to family barriers. Maghraby et al. (9) also showed that sexual harassment affected the private lives of nurses. Ali and Ezz El Rigal (16) reported that sexual harassment affected nursing students' family relationships. Gabay and Shafran Tikva (30) showed that sexual harassment led to a lack of protection, loneliness, and alienation (30), fear of being blamed by family, and fear of having restricted independent mobility. Sexually harassed victims refuse to disclose sexual harassment because they are afraid of disrupting their family relationships. Another reason is that most studies focus on the prevalence of sexual harassment rather than its reporting. However, the present study paid special attention to nurses' reasons for silence on sexual harassment in the workplace. Another reason could be the cultural contexts of different societies and the sensitivity of individuals to reporting the negative consequences of sexual harassment, so that sexual harassment did not have a clear definition for some nursing students, and they accepted it as part of their work (31). Sixty percent of the nurses had not reported sexual harassment (16). A review of the literature shows that studies have focused less on the effects of sexual harassment on nurses' family relationships. Their reasons may be fear of damaging the victims' private aspects and their special attention to the family. Iran is a family-oriented country and many studies have shown the effects of family on the work of Iranian nurses (32), so future studies must pay more attention to family barriers to disclosing sexual harassment in the workplace.

### Personal barriers

Nurses participating in the study were reluctant to disclose sexual harassment because of personal barriers. Adikaram et al. (18) in Sri Lanka found that self-control and self-discipline caused women to avoid using the term “sexual harassment” because of fear of social censorship, self-blame, and others' blame. Gilligan and Akhtar reported that in Asia, reports of sexual harassment were fewer than elsewhere because Asian women's fears of cultural feedback, in combination with izzat (honor/respect), haya (modesty), and sharam (shame/embarrassment), had a significant impact on disclosing sexual harassment (33). Some studies have also shown that sexually harassed nurses have psychological problems that can affect the disclosure of sexual harassment. Kim et al. (15) showed that sexually harassed nurses experienced severe fear and negative emotions. Tollstern Landin et al. (27) found that more than 30% of the sexually harassed nurses were reluctant to return to their workplace, and more than 40% of them expressed feelings of anger and fear. These findings show that nurses' sexual harassment, regardless of the type of different societies and cultures, causes devastating psychological effects on nurses, so they cannot trust others. According to Maghraby et al. (9), sexual harassment causes frustration, loss of trust in others, and loss of self-confidence among nurses. Therefore, personal barriers to disclosing sexual harassment must be reduced. To find and stop sexual harassment, it is best to keep an eye on the personal and individual problems of nurses and use primary prevention measures, such as professional psychological counseling.

Numerous studies have reported the incidence of violence in the nursing system. The high rate of physical violence is well known, and many organizations have focused on reducing violence at work rather than sexual harassment reports. However, research has not yet determined whether the low rate of reported sexual harassment is due to fewer reports or not (2). Failure to report is a major obstacle to eradicating sexual harassment from the healthcare and nursing systems. Nurses feel uncomfortable sharing the incidents they have encountered and disclose sexual harassment less frequently (23). First, nurses must report sexual harassment. Second, factors affecting disclosure of sexual harassment, including increasing knowledge and awareness (27), early preparation (34), self-care (15), support of sexually harassed victims (27), accurate reports (31), the existence of a systematic, fast, and decisive reporting system (15, 31), the establishment of security systems and policies to punish perpetrators (16), and effective coping strategies in nursing (15) are necessary. Employers must be careful to prevent and address sexual harassment as soon as possible. An action plan, including policies and staff training, must be implemented. Primary prevention is the first line of defense against sexual harassment (23). Measures must be taken to stop sexual harassment, such as sharing the issue, expressing strong opposition, imposing appropriate sanctions, informing employees of their right to report harassment, and developing ways to make everyone aware of their responsibilities (35).

## Limitations

The present study was conducted in southeastern Iran, where areas of religious sensitivity may have prevented participants from disclosing all of their personal information. Therefore, future studies should take into account the differences in cultural and ethnic backgrounds of Iranians when generalizing their findings. This study did not consider personal, social, and political factors such as personality, life experience, or hospital organizational structure. Therefore, similar studies should be conducted in other parts of the world to investigate these factors to gain further insight into why nurses do not disclose sexual harassment in the workplace. Also, it is suggested that studies be done on how employees feel, what they think, what they have experienced, and how they react to sexual harassment at work. These things can help keep sexual harassment from coming to light.

## Conclusion

In this study, nurses' reasons for not disclosing sexual harassment at work are explored, which has received little attention in the past. This study found that nurses did not speak out about sexual harassment because of fear of social stigma, organizational and legal barriers, family barriers, and personal barriers. Through improved education, creating a clear and specific system for reporting sexual harassment, and supporting laws and policies for nurses, managers, and policymakers can solve some of the reasons for nurses not disclosing sexual harassment.

## Data availability statement

The raw data supporting the conclusions of this article will be made available by the authors, without undue reservation.

## Ethics statement

The studies involving human participants were reviewed and approved by Kerman University of Medical Sciences. The patients/participants provided their written informed consent to participate in this study.

## Author contributions

MZ, PM, and MD designed the study and collected data. MZ, MAZ, PM, and MD contributed to the study design, they provided critical feedback on the study and qualitative analysis, and inputted to the draft of this manuscript. MZ and MAZ wrote the manuscript. All authors have read and approved the final manuscript.

## Conflict of interest

The authors declare that the research was conducted in the absence of any commercial or financial relationships that could be construed as a potential conflict of interest.

## Publisher's note

All claims expressed in this article are solely those of the authors and do not necessarily represent those of their affiliated organizations, or those of the publisher, the editors and the reviewers. Any product that may be evaluated in this article, or claim that may be made by its manufacturer, is not guaranteed or endorsed by the publisher.
